# Self-inflicted burns in Brazil: systematic review and meta-analysis

**DOI:** 10.1590/0100-6991e-20243665-en

**Published:** 2024-04-17

**Authors:** JOÃO HENRIQUE FONSECA DO NASCIMENTO, BENJAMIM MESSIAS DE SOUZA, SELTON CAVALCANTE TOMAZ, ADRIANO TITO SOUZA VIEIRA, MARINHO MARQUES DA SILVA, ANDRÉ BOUZAS DE ANDRADE, DIOGO RADOMILLE DE SANTANA, ANDRÉ GUSMÃO-CUNHA

**Affiliations:** 1 - Universidade do Estado da Bahia (UNEB), Departamento de Ciências da Vida - Salvador - BA - Brasil; 2 - Universidade Federal da Bahia (UFBA), Faculdade de Medicina da Bahia (FAMEB) - Salvador - BA - Brasil

**Keywords:** Burns, Brazil, Self-Injurious Behavior, Systematic Review, Meta-Analysis, Queimaduras, Brasil, Revisão Sistemática, Metanálise

## Abstract

**Introduction:**

burns represent a pivotal component of trauma in Brazil, accounting for 2 million incidents and 2,500 deaths annually. Self-intentional burns are associated with a worse prognosis, larger burned surface area, higher infection rates, and death. The lack of studies on the issue of self-immolation raises epidemiological questions regarding Brazilian victims. This study aimed to investigate the profile of burn events associated with self-injurious behavior among Brazilian victims.

**Methods:**

this systematic review was performed according to PRISMA 2020 guidelines and evaluated the correlation between self-injurious behavior as a cause of burns in Brazilian victims and its epidemiological implications in the last 20 years (2003-2023). The MeSH terms “Burns”, “Self-Injurious Behavior”, “Epidemiology” and “Brazil” were queried in the PubMed/MEDLINE, SciELO, and Cochrane Library databases, and, after selection by inclusion/exclusion criteria, the most relevant studies were critically analyzed.

**Results:**

From 1,077 pre-selected studies, 92 were potentially eligible, resulting in 7 manuscripts incorporated in this review. From 3,510 burned victims assembled in the pool of selected studies, 311 cases displayed self-injurious behavior. Burned patients who attempted to burn their lives have a higher risk of death (p<0.05; RR=5.1 [3.2-8.1]) and larger burned surface area (p<0.05; MD=19.2 [10-28.2]), compared to accidental cases. Moreover, the female gender was at a higher risk of attempting self-immolation (p<0.05; RR=4.01 [2.9-5.5]).

**Conclusion:**

our results show that self-inflicted burn cases were associated with a larger burned surface area and a higher risk of death, and the female gender was identified as a relevant risk factor in Brazil.

## RESUMO

**Introdução:**

Queimaduras representam um componente fundamental do trauma no Brasil, sendo responsáveis por 2 milhões de incidentes e 2.500 mortes anualmente. Queimaduras autointencionais estão associadas a pior prognóstico, maior superfície corporal queimada, maiores taxas de infecção e morte. A falta de estudos sobre a problemática da autoimolação levanta questões epidemiológicas em relação às vítimas brasileiras. O estudo se objetivou investigar o perfil das queimaduras associados ao comportamento autolesivo entre vítimas brasileiras.

**Métodos:**

Esta revisão sistemática foi realizada de acordo com as diretrizes PRISMA 2020 e avaliou a correlação entre comportamento autolesivo como causa de queimaduras em vítimas brasileiras e suas implicações epidemiológicas nos últimos 20 anos (2003-2023). Os termos MeSH “Burns”, “Self-Injurious Behavior”, “Epidemiology” e “Brazil” foram elencados no PubMed/MEDLINE, SciELO e Cochrane Library e, após seleção por critérios de inclusão/exclusão, os estudos mais relevantes foram analisados criticamente*.*

**Resultados:**

Dos 1.077 estudos pré-selecionados, 92 foram potencialmente elegíveis, resultando em 7 manuscritos incorporados nesta revisão. Das 3.510 vítimas queimadas reunidas no conjunto de estudos selecionados, 311 casos apresentaram comportamento autolesivo. Pacientes que tentaram autoimolação apresentam maior risco de morte (p<0,05; RR=5,1 [3,2-8,1]) e maior superfície corporal queimada (p<0,05; MD=19,2 [10-28,2]), em comparação com casos acidentais. Ademais, o sexo feminino apresentou maior risco para tentativa de autoimolação (p<0,05; RR=4,01 [2,9-5,5]).

**Conclusão:**

Nossos resultados mostram que os casos de queimaduras autoprovocadas foram associados a uma maior área de superfície corporal queimada e a um maior risco de morte, e o sexo feminino foi identificado como um fator de risco relevante no Brasil.

## INTRODUCTION

Burns are a pivotal epidemiological component of trauma^1^ ^-^ ^3^. Worldwide reports compiled an average of 265,000 annual burn-related deaths and approximately 11 million burn victims demanding specialized care per annum, setting these events as the 4^th^ leading cause of direct trauma globally^3^ ^,^ ^4^. According to official data from the Brazilian Ministry of Health, burns represent approximately 2 million occurrences and 2,500 fatalities yearly in Brazil^5^ ^,^ ^6^. The impacts of burns extend beyond mortality, as they might result in critical physical and psychological sequelae and compromise victims’ social interactions and labor potentials to varying degrees^7^ ^,^ ^8^. 

These events can be categorized separately into unintentional/accidental or intentional cases, given the particularities of the triggering circumstances of each cluster, and the group of intentional cases can also be subdivided into aggression or self-inflicted burns^9^ ^,^ ^10^. Intentional self-inflicted burns comprise a part of the self-injurious behavior, ranging from self-flagellation to the suicide attempt itself^9^ ^,^ ^10^. Although less frequent among trauma prevalence in Brazil, the suicidal patient by self-immolation, signifying the act of self-sacrifice by fire, has a relevant social presence, intense emotional burden, and particular impact on morbimortality, and, in many cases, the health professional team does not fully comprehend the singularities of these experiences^1^ ^,^ ^11^. Previous studies have demonstrated that burns due to self-sacrifice attempts are associated with poor prognosis, larger burned body surface area, frequent wound infections and sepsis, and higher mortality rate, especially when the patient presents pre-existing psychiatric diagnoses, such as depression or psychotic disorders^2^ ^,^ ^9^ ^,^ ^12^. Several authors have shown a substantial role of sociocultural aspects linked to these incidents, vastly correlated with gender, socioeconomic vulnerability, and religiosity^1^ ^,^ ^2^ ^,^ ^13^ ^-^ ^16^. These associations are prominent in Asian countries, such as India, Iran, and Iraq, where the prevalence of burns of this nature is exceedingly high^1^ ^,^ ^2^ ^,^ ^9^ ^,^ ^15^ ^-^ ^18^. 

Therefore, given the pertinence of the theme and the paucity of studies on Brazilian victims, we conducted a systematic review with meta-analysis on the phenomenon of intentional self-inflicted burns and the epidemiological impacts on morbidity and mortality in Brazil.

## METHODS

### Search strategy

The present study is a systematic review on victims of intentional self-inflicted burns in Brazil. To guide the investigation, the authors adopted the steps for a systematic review construction recommended by Cochrane^19^. The performance of the review also followed the Preferred Reporting Items for Systematic Reviews and Meta-Analyses (PRISMA) 2020 protocol^20^ ^,^ ^21^. Two independent and qualified reviewers queried the most renowned scientific databases: PubMed/MEDLINE, SciELO, and the Cochrane Library. The Medical Subjects Heading (MeSH) terms “Burns”, “Self-Injurious Behavior” and its entry term “Self-Injury”, “Epidemiology”, and “Brazil” were revised and chosen according to the PECO method for systematic reviews (Table 1), with the equivalents Health Sciences Descriptors (Descritores em Ciências da Saúde - DeCS) terms in Portuguese “Queimaduras”, “Comportamento Autodestrutivo” and “Autolesão”, “Epidemiologia”, and “Brasil”. The search was restricted to studies conducted in the Brazilian population and published in English or Portuguese between January 2003 and January 2023, encompassing two decades of the most recent literature. For the search strategy, the chosen keywords were queried individually and combined using the Boolean operators “AND” and “OR”. Table 2 shows the specific search strategy for the electronic databases.

**Table 1:** Research descriptors according to the PECO method for systematic reviews.

Problem	Self-Injurious Behavior AND Brazil
Exposure	Burns
Comparison	Self-injury Intentional Burns OR Accidental Burns
Outcome	Death OR Survival

**Table 2:** Search strategy for Electronic Databases.

Search 1	“Burns”, “Queimaduras” (combined by “OR” operator)
Search 2	“Self-Injurious Behavior”, "Self-Injury", “Comportamento Autodestrutivo”, “Autolesão” (combined by “OR” operator)
Search 3	“Epidemiology”, “Epidemiologia” (combined by “OR” operator)
Search 4	“Brazil”, “Brasil” (combined by “OR” operator)
Final Search	Search 1 AND/OR Search 2 AND/OR Search 3 AND/OR Search 4

Hoy’s risk of bias tool^22^ was applied to evaluate the quality and risk of bias among the studies and considered suitable and easy to use in this review. The tool comprises 10 items that assess the external (items 1-4) and internal (items 5-10) validity of prevalence studies and covers four bias domains: (1) selection-related bias, (2) bias associated with non-response, (3) measurement-related bias, and (4) bias associated with analysis. After completing the judgment, the reviewer chose one alternative among two: (“yes”: 1 point) low risk or (“no”: 0 points) high risk. The high-risk option was chosen if the study lacked basic information to make the judgment. The overall quality score was rated as low (8 to 10 items checked as high-risk/”yes”), moderate (5 to 7 items checked as high-risk/”yes”), and high (0 to 4 items checked as high-risk/”yes”) risk of bias.

### Eligibility criteria

The following criteria were used to consider a study eligible for general analysis:

1. Original studies with adult populations on intentional self-injurious burns or attempted suicide by burns compared to accidental self-inflicted burns and event-related patient outcomes;
2. Studies placed on Brazil and/or performed with Brazilian populations;
3. Article type including classical article, comparative study, evaluation study, multicenter study, observational study, and ecological study.

Publications were excluded if: full texts were not accessible, there was no discrimination between groups of intentional and accidental burns, they were duplicated, and the study did not display a design that matched our objectives.

### Data extraction

Two reviewers independently screened papers titles and abstracts, and if they met the eligibility criteria, the full text was read and included in the sample set. Any disagreement over the study selection between the two reviewers was resolved through consensus or consultation with a third or fourth reviewer. The retrieved data included: title of the study, first author, year, design of the study, place of study, study period, the total number of cases, the intention of the incident (accidental or intentional), the total number of fatalities, the in-hospital mortality rate (TMH) and intention-related deaths, gender, mean age, etiology/agents of burns, burn accelerators, burned body surface area, and airway injury.

### Statistical analyses

Overall results were presented as weighted mean ± weighted standard deviation. The data acquired from the studies were analyzed in terms of relative risk/risk ratio (RR) for dichotomous variables or mean differences (MD) for continuous variables, considering a confidence interval greater than 95% (p<0.05). All statistical analyses and graph generation were carried out in the R-studio program (R Foundation, v.4.0.3). Heterogeneity was assessed by the Q test and I² statistic, of which an I² >50% was considered a substantial level of heterogeneity, I² between 25% and 50% was considered moderate, I² <25% was interpreted as no evidence of relevant heterogeneity, with all correlated with p<0.05 to indicate significant differences. A random-effects model (Mantel-Haenszel method) was chosen to perform the meta-analysis, including in the presence of statistical heterogeneity or whether the situation was identified as bearing potential for heterogeneity, which is recommended for adjusting the observed variability in case of high heterogeneity^1^ ^,^ ^19^ ^,^ ^23^. The subgroup meta-analysis was performed with variables in which sufficient studies were available to form subgroups with two studies or more.

## RESULTS

**Figure 1:**
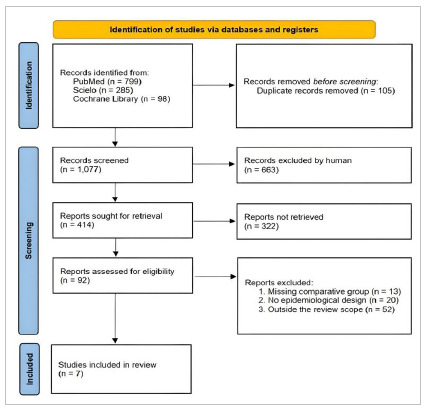
Flow chart of the search method and data extraction.

The initial quest identified 1,182 publications from all databases, of which 105 duplicated records were excluded, recovering 1,077 manuscripts. Six hundred sixty-three (n=663) records were excluded after title screening, and three hundred and twenty-two (n=322) publications were ruled out after abstract assessment, resulting in 92 potentially relevant papers. After careful revaluation of the abstracts and full-text reading of the retrieved publications that met the eligibility criteria, seven^4^ ^,^ ^10^ ^,^ ^11^ ^,^ ^13^ ^,^ ^24^ ^-^ ^26^ (n=7) manuscripts composed the final sample, which underwent qualitative and quantitative analyses (Figure 1). The overall risk of bias of the 7 included studies was moderate (average score=5.86 points; SD=0.69). None of the studies were at low or high risk of bias. The items that most varied between studies and that seemed to impose a risk of bias were two criteria of internal validity (item 5 and 9) that focus on measurement bias and analysis bias. These biases were related to the length of the shortest prevalence period and collecting data directly from the subjects (as opposed to a proxy). Moreover, all studies were at low risk of bias for most criteria of internal validity (item 1 to 3).

The selected original articles were longitudinal studies, and the slight majority were prospective (57.1%). The locations of the studies were geographically distributed among the Southeast (28.5%), Midwest (28.5%), and South (43%) regions of Brazil between 2003 and 2013. The seven selected manuscripts were aligned with the central objective of this study, which was, besides providing the total number of burn victims, to differentiate between accidental and self-inflicted burns (suicide attempts). Table 3 shows the core information obtained from the selected articles. Attempted murder (assault) burn victims were excluded from the analyses. 

**Table 3:** Selected studies for the systematic review.

Author	Year	Study design	Study place	Period of study	Total of patients	Number of accidental burns	Number of self-inflicted intentional burns
Macedo et al.^24^	2007	Longitudinal and prospective	Brasília/DF	2004 - 2005	278	263	15
Macedo et al.^13^	2011	Longitudinal and prospective	Brasília/DF	2008 - 2009	278	263	15
Leão et al.^25^	2011	Longitudinal and prospective	Belo Horizonte/MG	2009 - 2010	687	605	82
Campos et al.^26^	2014	Longitudinal and prospective	São Paulo/SP	2005 - 2010	163	131	32
Queiroz et al.^4^	2015	Longitudinal and retrospective	Londrina/PR	2010 - 2012	293	236	35
Duarte et al.^10^	2015	Longitudinal and retrospective	Porto Alegre/RS	2003 - 2012	1,633	1,519	114
Mireski et al.^11^	2016	Longitudinal and retrospective	Londrina/PR	2011 - 2013	178	129	19
				Total	3,510	3,146	311

[Table t4]

**Table 4 t4:** Hoy’s risk of bias tool.

	Was the study’s target population a close representation of the national population in relation to relevant variables?	Was the sampling frame a true or close representation of the target population?	Was some form of random selection used to select the sample, OR was a census undertaken?	Was the likelihood of nonresponse bias minimal?	Were data collected directly from the subjects (as opposed to a proxy)?	Was an acceptable case definition used in the study?	Was the study instrument that measured the parameter of interest shown to have validity and reliability?	Was the same mode of data collection used for all subjects?	Was the length of the shortest prevalence period for the parameter of interest appropriate?	Were the numerator(s) and denominator(s) for the parameter of interest appropriate?	Rate
Macedo et al.^24^	N	N	N	Y	Y	Y	Y	Y	N	Y	M
Macedo et al.^13^	N	N	N	Y	Y	Y	Y	Y	N	Y	M
Leão et al.^25^	N	N	N	Y	Y	Y	Y	Y	N	Y	M
Campos et al.^26^	N	N	N	Y	Y	Y	Y	Y	Y	Y	M
Queiroz et al.^4^	N	N	N	Y	N	Y	Y	Y	N	Y	M
Duarte et al.^10^	N	N	N	Y	N	Y	Y	Y	Y	Y	M
Mireski et al.^11^	N	N	N	Y	N	Y	Y	Y	N	Y	M

Y: yes; N: no; H: high risk of bias; M: moderate risk of bias; L: low risk of bias

A total of 3,510 burn victims were assembled in the pool of selected studies, comprised of 2,370 (67.52%) males and 1,140 (32.48%) females, and displaying an overall proportion of 2 men for each woman. The mean age was 27.8 (±29.7) years, the mean total body surface area burned was 20.1% (±30.1%), and the mean in-hospital mortality rate was 15.77%. One thousand one hundred and ninety (n=1,190) patients were examined for airway injuries, and 261 cases were positive for lesions, representing an average of 25.4% (±21.4%) of victims among the studies. Direct flames/fire burns were the most frequent cause of the incidents, accountable for 2,077 (59.2%) victims, followed by scalding (938 victims; 26.7%), electricity (289 victims; 8.2%), chemical agents (45 victims; 1.3%), and others (48 victims; 1.4%). One hundred and thirteen (n=113; 3.2%) victims did not have information about their used burn agent. Five hundred and thirty (n=530) victims had the type of accelerator assessed, and liquid alcohol computed the highest number of cases (n=470; 88.7%), followed by gasoline (n=28; 5.3%) and high-voltage electricity (n=11; 2.1%), although 21 (3.9%) cases did not explicitly provide information on the used accelerator.

**Figure 2:**
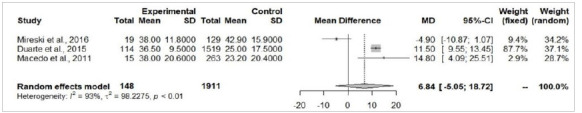
Forest Plot of the age analysis between self-inflicted (experimental) and accidental burn victims (control).

As previously stated, homicide attempts were excluded from the universe of patients (n=57), resulting in a total of 3,457 cases, of which 311 (9%) were due to self-inflicted intentional burns and 3,146 (91%) cases were accidental burns. There was no difference concerning age between accidental and self-inflicted burn victims (MD=6.84; 95%-CI=-5.05-18.72; p=0.25; Figure 2). Most self-inflicted burn patients were female (64.9% female and 35.1% male), with an average female:male ratio of 2:1. 

**Figure 3:**
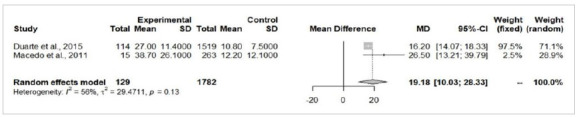
Forest Plot of the burned body surface area analysis between self-inflicted (experimental) and accidental burn victims (control).

**Figure 4:**
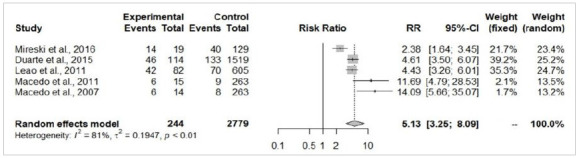
Forest Plot of the risk of death analysis between self-inflicted (experimental) and accidental burn victims (control).

**Figure 5:**
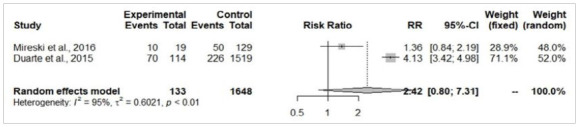
Forest Plot of the risk of airway injury analysis between self-inflicted (experimental) and accidental burn victims (control).

The patients from the self-extermination attempted group presented a significantly larger total body surface area burned (MD=19.18%; 95%-CI=10.03-28.33; p<0.05; Figure 3) and higher risk of death (RR=5.13; 95%-CI=3.25-8.09; p<0.05; Figure 4). There were no significant differences in the risk of airway injury between accidentally burned patients and cases of self-immolation (RR=2.42; 95%-CI=0.8-7.31; p=0.11; Figure 5). From the perspective of gender, male patients were more prevalent in accidental burn events (71.6%), yet females were more prominent among the victims of attempted self-immolation (64.9%). Indeed, men presented a statistically significant higher risk of being victims of burns by accident (RR=1.32; 95%-CI=1.10-1.57; p<0.05; Figure 6A), whereas suicide attempts were statistically more frequent among women (RR=4.01; I95%=2.94-5.46; p<0.05; Figure 6B). These findings align with the observation that a hospitalized burned woman exhibited a significantly higher chance of being a victim of a suicide attempt than an accidental burn (RR=2.46; 95%-CI=2.00-3.04; p<0.05; Figure 7).

**Figure 6A:**
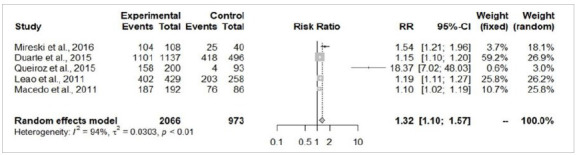
Accidental burn cases between men (experimental) and women (control).

**Figure 6B:**
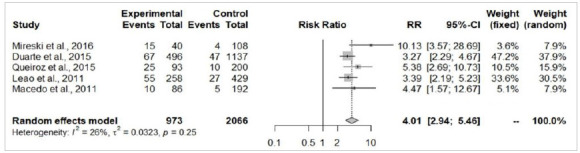
Self-inflicted burn cases between women (experimental) and men (control).

**Figure 7:**
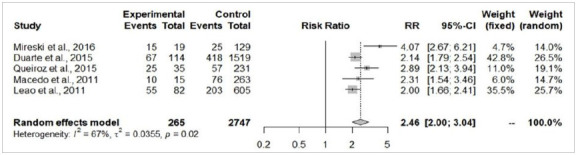
Comparison between hospitalized women, victims of burns by attempted suicide (experimental) and accidental burn (control).

## DISCUSSION

This systematic review and meta-analysis focused on reviewing and summarizing the epidemiological features, prognosis, and risks related to the association of burns and self-injurious behavior in Brazil and conducting a statistical evaluation of the published studies examining Brazilian victims. The result of the meta-analysis of 7 studies evinced that the overall pooled prevalence of males was more prominent (67.2%), victims were young adults (average age of 27.8 years), and cases were classified as major burns according to the average total body surface area burned (20.1%). The result of subgroup analysis based on the intention of the burn event (accidental vs. intentional self-inflicted burn) showed that the attempted self-immolation was associated with a larger total body surface area burned (MD=19.18%; p<0.05), a higher risk of death (RR=5.12; p<0.05), and a higher chance of victim being a woman (RR=4.01; p<0.05).

Burns remains a severe and global public health issue^2^ ^,^ ^3^ ^,^ ^9^ ^,^ ^27^. In Brazil, epidemiological data from government databases indicate an annual incidence in units of millions to represent these victims, whose main risk factors lie in liquid alcohol and gasoline handling, fireworks, bonfires, socioeconomic vulnerability, and interpersonal violence^6^. The present review showed that direct flames still represent the principal cause of burns among Brazilian victims, responsible for 2,077 (59.2%) cases in the sample set. Direct flames also have been shown to lead the burn agent list of causes in several previous studies, regardless of the used accelerator, not only for self-inflicted cases but also for non-intentional incidents^3^ ^,^ ^6^ ^,^ ^27^. 

Liquid alcohol persists as one of the most predominant accelerators used by victims, accounting for 88.7% of the cases in this review. Several authors have yielded similar results in previous reports, highlighting that liquid alcohol is a frequent and common trigger of burns in many countries^3^ ^,^ ^6^ ^,^ ^8^ ^,^ ^27^ ^,^ ^28^. In this matter, it is common knowledge that Brazil has invested in public policies to replace liquid alcohol with gel alcohol for widespread use in the past decades. Despite the regulatory norms for the home use of alcohol published by the National Health Surveillance Agency (Agência Nacional de Vigilância Sanitária - ANVISA) in 2002, which addresses the prohibition of the 54ºGL (Gay-Lussac) alcohol commercialization, the “common” alcohol is still easily acquired by the general Brazilian population^28^ ^,^ ^29^. Moreover, it was only in 2013, through a Government Resolution, that the manufacture and distribution of 54ºGL liquid alcohol were prohibited in the national territory^29^. 

Culturally, most Brazilian homes still dispose of liquid alcohol nowadays for antiseptic use, house cleaning, as fuel in barbecues, to preserve several food items from spoiling faster for some families, as an ingredient in home remedies and informal treatments, which facilitates accidents, as reported by Queiroz et al.^4^ ^,^ ^28^. Colloidal alcohol use is encouraged because it presents lower volatility and lower combustion potential than traditional liquid alcohol, without losing bactericidal efficiency, according to Arrunátegui et al., an attribute that makes the use of this type of alcohol safer^28^ ^,^ ^29^. Undoubtedly, the COVID-19 pandemic and the popularization of alcohol use as a sanitizer agent (even at home) contributed to increasing alcohol-related burn incidence in the past few years^30^. The at-home practice of hand sanitizing using 70% alcohol has remarkably spread since 2020 and, in turn, has culminated in the rise of accidental household burns incidence caused by this new hygiene habit, which perpetuated from the quarantine period to the present day, as briefly reported by Hohl, who showed that cases of alcohol-related burns during the COVID-19 outbreak in Brazil resulted in serious victims with up to 40% of burned body surface area and almost 40% of these cases needed surgical debridement and skin coverage graft^30^ ^,^ ^31^.

From the perspective of violence, self-inflicted intentional burns encourage and stimulate a closer look. Suicide is a relevant component in the daily routine of trauma and emergency services in Brazil, where, despite the significant 29.4% incidence growth between 1996 and 2016, there is still a scarcity of scientific production and public policies in several nuances of this particular issue^32^, for instance, self-inflicted burns. Burns by intentional self-harm do not exhibit an official high prevalence in Brazil, indeed. Yet, these events are not uncommon and are always present in emergency services nationwide, whose victims have particular features, making their management challenging and their prognosis obscure^13^. Conversely, this aforementioned low prevalence might reflect a failure in notification by public health professionals and services, which, ultimately, might imply that intentional self-inflicted burns as part of self-injurious behavior are possibly underreported in Brazil. 

This review evinced that burn victims with self-harm intention exhibited a larger total body surface area burned (MD=19.2% [10.03-28.33]) and a higher chance of death (RR=5.13 [3.25-8.09]). Duarte et al. demonstrated that suicidal burned patients have higher mortality rates, larger burned body surface area, higher rates of complications, and worse prognosis than victims of accidental incidents^10^. Macedo et al. investigated the particularities regarding burn victims of suicide attempts and also demonstrated that these victims have larger burned body surface area and higher in-hospital mortality rates^13^. Previous studies have shown the factors that might directly or indirectly influence the outcome of the suicidal burned victim, identifying positive associations with the presence of deeper burn lesions, higher rates of wound infection, higher rates of colonization by multidrug-resistant bacteria, previous suicide attempts, a higher number of prior psychiatric diagnoses and pre-existing abuse of alcohol and/or other drugs^10^ ^,^ ^13^ ^,^ ^24^.

Gender seems to pose differences in epidemiological and clinical characteristics of victims in the present review. Our results showed that males appear to be victims of burns more frequently, both in the overall total number of events, of which men were responsible for 67.5% of the victims, and also in the accidental burn group, in which men accounted for 71.6% of the cases. Many sociocultural factors in Brazil support this finding, for instance, greater male exposure to flames, fire, and bonfires, a common habit of ignoring exposure risks (such as firework handling), and more frequent burn accidents related to work activities among Brazilian men^3^ ^,^ ^4^. On the other hand, the female gender was predominant regarding burn victims due to suicidal behavior, computing 64.9% of the cases. The overall panorama suggests that Brazilian men are more often victims of burns in general, while women are accountable for most suicide attempts by burns, corroborated by our meta-analysis. Further, by assessing the pool of hospitalized burned females, the results evidenced that this Brazilian woman exhibited an approximately 140% higher risk of being admitted due to a suicide attempt than an accidental burn. 

Female sex has been implied as a risk factor for attempting self-immolation worldwide^1^ ^,^ ^14^ ^,^ ^17^ ^,^ ^33^. Ahmadi et al. demonstrated that women are the predominant victims of attempted self-sacrifice by fire in Iran, where the self-immolation method sums up 40% of cases, of which women compute between 70 and 88% of all these victims, and the female mortality rate reaches up to 80%^14^. Maghsoudi et al. assessed 412 intentional self-inflicted burn incidents in Iran between 1998 and 2002, and 99% of all cases were women^33^. Mabrouk et al. investigated gender in a study with 759 Egyptian burn victims, and 91.3% of the suicidal behavior cases were females^34^. In Kurdistan, a report on the female-to-male ratio of self-immolation attempters showed a proportion of 10 to 1^1^. At the time of writing, no prior systematic review or meta-analysis evaluated the differences between victims of accidental burns and those related to self-injurious behavior in Brazil, and the present investigation revealed a proportion of 2 women for each man in the suicide attempt by intentional burning, emphasizing gender as a matter of relevant concern in the epidemiology of this trauma nationwide.

Attempted suicide by means of burns is an uncommon type of suicidal behavior in Western nations, such as the United States and western European countries, where it totals approximately 1% of all forms of suicide^2^ ^,^ ^14^. Developing countries with a population-wide poor level of education exhibit higher frequencies of self-injurious behavior^1^ ^,^ ^32^ ^,^ ^35^. Low- and middle-income countries register 80% of all global suicide, also recording a higher prevalence of self-immolation, which places Brazil at greater risk^1^ ^,^ ^11^ ^,^ ^14^ ^,^ ^32^. 

Campos et al. examined gender differences in a five-year observational study with 163 burned patients from the state of São Paulo, and 54% of the female deaths were due to suicide, whereas only 21% of deaths in the male group were related to self-extermination^26^. Moreover, Ramim et al. explored suicidal behavior and marital status in a 35-patient study of victims of self-immolation in Iran and concluded that wives who attempted to burn their lives were young and living in a traditional environment^17^. Marital status and possible spousal problems are pertinent variables for the scope of this review since domestic violence often pushes women to self-harm by burning, as reported by Diniz et al. in a 35-hospitalized burned women study in Salvador (Brazil), which showed that 83% of victims suffered some degree of domestic violence and 100% of women who attempted self-extermination by burns declared that the violence inflicted by their husband or partner preceded their attempt of suicide^36^. Nonetheless, age did not exhibit a significant difference between groups in our sample, and the variable marital status was not available for analysis, which is one of the limitations of this review.

The psychological state of mind of the victims is another attribute that needs to be carefully explored. Certainly, self-extermination by direct flames is one of the most violent and dramatic forms of suicidal behavior, which may emphasize the extreme emotional disturbance and psychological suffering of victims^2^ ^,^ ^10^ ^,^ ^17^ ^,^ ^33^ ^-^ ^35^. Paradoxically, the psychological factor seems to be both associated with worse outcomes and, on the other hand, correlated with higher chances of survival since many of these occurrences may lack premeditation or actual intention of death, which, in many cases, result in a risk of death similar to accidental burns^10^. As published by Natarajan in a study in India, many of the 168 (65%) female victims of self-inflicted burns revealed that they had no actual intention of achieving death, yet they indeed wanted to express their extreme psychological and emotional anguish^18^. Duarte et al., when evaluating 1,734 cases in the state of Rio Grande do Sul, showed that psychological distress was significantly correlated with the suicidal behavior among burn victims, in which a prior psychiatric diagnosis was present in approximately 50% of these patients, much higher than the 3.4% of previous psychiatric diagnoses seen in the accidental burn group^10^.

This review with meta-analysis holds some limitations. The authors aimed to investigate the epidemiological features of self-inflicted burns nationwide, yet the absence of manuscripts from some parts of Brazil precludes a broader analysis. The scarcity of data regarding the North and Northeast regions is challenging since the methodological strategy for reports acquisition could not retrieve articles from these regions to compose the final sample unit. Moreover, the possible existence of sociocultural discrepancies between these regions and the rest of the country might hold relevant differences that could contribute to our analysis regarding access to health services, risk of burns, whether or not there are burn unit centers, and vital information on the profile of the burned victims^3^. In these regions, it is essential to consider burns from fireworks and bonfires due to cultural, religious, and partying traditions^3^ ^,^ ^6^ ^,^ ^27^, for instance, June celebrations and Saint John traditional festival in the Northeast and the folk festivals in the North. Additionally, level of education, marital status (as already mentioned in this discussion), work occupation, income, previous psychiatric diagnoses, drug abuse, prior attempts, family support network, access to private or public health services, and other socioeconomic vulnerabilities were not available for assessment, and all those variables might incorporate confounding factors that could have biased the analyses. Another point of concern that was assessed during the risk of bias analysis was related to the length of the studied period and the method of data acquisition, which were more likely to be associated with bias when a short time-frame was studied (e.g., 1 to 2 years) or whether data were collected retrospectively from patient’s medical records. These limitations constitute bases for vaster analytical investigations and primary studies by research groups, university hospitals, emergency and trauma centers, and government health departments regionwide and nationwide to generate more robust data on burns, especially those related to suicidal behavior. 

## CONCLUSION

In conclusion, this systematic review with meta-analysis leads to the inference that intentional self-inflicted burns in the scope of suicidal behavior represent a severe public health issue and lies within the core of the trauma epidemiology of burns in Brazil. These Brazilian victims are associated with a larger burned body surface area and a higher risk of death than accidental cases. Further, the female gender was identified as a considerable risk factor for these circumstances, posing Brazilian women as a central interventionist target for public policies and preventive campaigns.
